# Next-Generation mRNA Vaccines in Melanoma: Advances in Delivery and Combination Strategies

**DOI:** 10.3390/cells14181476

**Published:** 2025-09-22

**Authors:** Stefano Zoroddu, Luigi Bagella

**Affiliations:** 1 Department of Biomedical Sciences, University of Sassari, Viale San Pietro 43/b, 07100 Sassari, Italy; 2 Sbarro Institute for Cancer Research and Molecular Medicine, Centre for Biotechnology, College of Science and Technology, Temple University, Philadelphia, PA 19122, USA

**Keywords:** mRNA vaccines, melanoma immunotherapy, lipid nanoparticles (LNPs), intratumoral delivery, combination therapies

## Abstract

Messenger RNA (mRNA) vaccines have redefined cancer immunotherapy, offering unparalleled flexibility to encode tumor-specific antigens and to be adapted to individual mutational landscapes. Melanoma, with its high mutational burden and responsiveness to immune checkpoint blockade, has become the leading model for translating these advances into clinical benefit. Recent innovations in delivery—ranging from lipid nanoparticles and polymeric carriers to biomimetic hybrids and intratumoral administration—are dismantling long-standing barriers of stability, targeting, and immunogenicity. Clinical milestones, including the randomized phase IIb KEYNOTE-942, show that adding the personalized neoantigen vaccine mRNA-4157 (V940) to pembrolizumab prolonged recurrence-free survival versus pembrolizumab alone (HR 0.561, 95% CI 0.309–1.017; 18-month RFS 79% vs. 62%), with the ASCO 3-year update reporting 2.5-year RFS 74.8% vs. 55.6% and sustained distant metastasis-free survival benefit in resected high-risk melanoma. Parallel preclinical studies highlight the potential of multifunctional platforms co-delivering cytokines or innate agonists to reshape the tumor microenvironment and achieve durable systemic immunity. As artificial intelligence drives epitope selection and modular manufacturing accelerates personalization, mRNA vaccines may have the potential to transition from adjuncts to main therapies in melanoma and beyond.

## 1. Introduction

### 1.1. Overview mRNA Vaccines (Oncology Focus)

**Figure 1:**
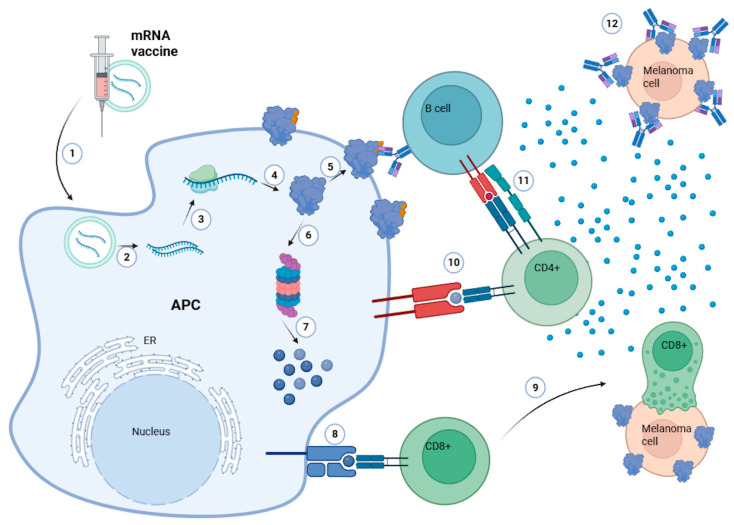
Mechanism of action of mRNA cancer vaccines and immune activation in melanoma. Following injection, lipid nanoparticle-formulated mRNA is taken up by antigen-presenting cells (APCs) (1,2), where it is released into the cytoplasm and translated into tumor-associated antigens or neoantigens (3). These antigens undergo proteasomal degradation (4–6) and are presented on major histocompatibility complex (MHC) class I and II molecules (7,8), leading to the activation of CD8^+^ cytotoxic T lymphocytes and CD4^+^ helper T cells (9,10). CD4^+^ cells further support B cell activation (11), resulting in the secretion of tumor-specific antibodies (12). This integrated cellular and humoral response targets melanoma cells expressing the encoded antigens, contributing to effective tumor eradication.

mRNA (messenger RNA) vaccines have rapidly evolved from a conceptual innovation to a transformative platform in modern medicine, with profound implications for cancer immunotherapy [1,2]. While the term “vaccine” is traditionally associated with preventive immunization against infectious diseases, in oncology it refers to therapeutic mRNA-based cancer vaccines—immunotherapy candidates designed to elicit or reinforce tumor-specific immune responses in patients with established malignancy. Their development has been catalyzed by the success of COVID-19 vaccines, which have demonstrated the feasibility, safety, and scalability of preventive mRNAs for an infectious disease globally [3]. Unlike DNA-based vaccines, mRNA vaccines do not require entry into the nucleus and avoid the risk of genomic integration, as translation occurs directly in the cytoplasm, leading to efficient and transient protein expression [4]. This feature makes them particularly suitable for oncology, where the timely induction of tumor antigen-specific immune responses is critical [1]. In cancer immunotherapy, mRNA vaccines work by encoding tumor-associated antigens or tumor-specific neoantigens, which are translated into proteins after cellular uptake and presented via major histocompatibility complex (MHC) class I and II molecules to activate cytotoxic CD8^+^ T cells and helper CD4^+^ T cells (Figure 1) [2,5,6,7].

This dual activation promotes both cellular and humoral immunity. While cellular responses are central to antitumor efficacy, humoral immunity may provide complementary benefits by broadening antigen recognition across heterogeneous tumor cell populations but is not universally required for clinical activity [4,8]. It is important to note that mRNA vaccines have been constructed to incorporate multiple epitopes in peptides of up to 1000 amino acids into a single construct, enabling broad immune coverage against tumors with high mutational burden such as melanoma [1,9]. Furthermore, their synthetic production allows for rapid customization based on patient-specific mutational profiles, facilitating the development of personalized cancer vaccines, a strategy that is entering phase III clinical trials (e.g., mRNA-4157/V940 in combination with pembrolizumab) with promising results in achieving partial preventing of melanoma recurrence [5]. Several technological advances underpin the clinical maturation of mRNA vaccines for oncology. The use of chemically modified nucleosides, such as pseudouridine or N^1^-methylpseudouridine, improves the stability and translation of transcripts while reducing innate immune recognition that could otherwise compromise protein expression [3]. Innovations in in vitro transcription methods have simplified large-scale production and improved transcript purity, minimizing double-stranded RNA contaminants that could trigger unwanted inflammatory pathways [4]. Furthermore, the advent of lipid nanoparticle (LNP) delivery systems has overcome a fundamental obstacle to the application of mRNA vaccines, protecting labile RNA from degradation, facilitating cellular uptake, and ensuring efficient endosomal escape [2,10,11]. From a therapeutic standpoint, mRNA vaccines occupy a unique niche in the immuno-oncology landscape. Their inherent mechanism of action allows for integration with other modalities, such as immune checkpoint inhibitors or cytokine therapies, to enhance antitumor responses and overcome immunosuppressive tumor microenvironments [5]. The specificity for the encoded antigens and epitopes, speed of production, and scalability of this platform position mRNA immunotherapy medicine as a promising pillar of future personalized cancer therapies [1].

### 1.2. Why Melanoma Is a Leading Target (Mutational Burden, Immunogenicity)

Melanoma is widely recognized as one of the most immunogenic human malignancies, a property largely attributable to its exceptionally high tumor mutational burden resulting from chronic exposure to ultraviolet radiation [12,13]. This extensive mutational burden generates a broad spectrum of tumor-specific neoantigens, which are aberrant peptide fragments uniquely expressed on malignant melanocytes and absent from normal tissues [12,14]. These neoantigens are processed and presented by major histocompatibility complex molecules, making melanoma cells particularly visible to the immune system and facilitating strong activation of cytotoxic and helper T cells [12,15]. The strong correlation between tumor mutational burden and immune response has been repeatedly confirmed in clinical and real-world studies [16,17]. Meta-analyses demonstrate that melanoma patients with high mutational burden are statistically more likely to experience improved overall and progression-free survival when treated with immune checkpoint inhibitors targeting programmed cell death protein 1 or cytotoxic T-lymphocyte-associated antigen 4, compared with patients harboring low mutational burden tumors [15,18]. Importantly, a high mutational burden increases the likelihood of generating immunogenic neoantigens, but this factor alone does not determine clinical benefit. The composition of the tumor microenvironment—particularly the degree of effector and memory T cell infiltration and the balance between pro-inflammatory and immunosuppressive populations—also plays a decisive role in shaping therapeutic outcomes [12,14]. As observed in other solid tumors, deregulation of oncogenic transcriptional programs has been shown to promote immune evasion and resistance to therapies, underscoring the importance of targeting these pathways in the design of mRNA-based immunotherapies [19,20,21]. The mutational heterogeneity of melanoma, frequently involving BRAF, NRAS, and NF1 alterations, further diversifies the antigenic landscape and increases the likelihood of generating highly immunogenic epitopes suitable for vaccine development [12,13]. Similar to insights gained from the characterization of microRNA-mediated regulatory networks in other solid tumors, these molecular signatures in melanoma can inform the selection of optimal targets for mRNA-based immunotherapy [22,23]. Recent trials highlight the translational relevance of these features: the phase IIb KEYNOTE-942 study demonstrated that combining a personalized mRNA-based neoantigen vaccine (mRNA-4157/V940) with pembrolizumab significantly reduced recurrence rates in patients with resected high-risk melanoma, providing compelling clinical evidence that leveraging tumor-specific mutational profiles can enhance antitumor immunity beyond checkpoint inhibition alone [15,24]. Collectively, melanoma’s ultraviolet-driven mutation spectrum, high neoantigen density, and pre-existing T-cell reactivity establish it as an optimal model for testing and refining mRNA vaccine platforms [12,13]. These intrinsic biological properties not only facilitate the generation of personalized vaccines but also allow for synergistic integration with existing immunotherapies, supporting the continued prioritization of melanoma in vaccine-based clinical research [12,15].

## 2. mRNA Vaccine Design and Targets in Melanoma

### 2.1. Tumor-Associated vs. Tumor-Specific Neoantigens

Tumor antigens are broadly classified into two categories: tumor-associated antigens and tumor-specific neoantigens [25,26]. Tumor-associated antigens (TAA) are self-proteins that are normally expressed at low levels in adult tissues but are aberrantly overexpressed or re-expressed during malignant transformation, such as cancer-testis antigens or lineage-restricted melanocyte proteins including MART-1 and tyrosinase [27,28,29]. Although these antigens are shared across patients and amenable to “off-the-shelf” vaccine designs, their presence in normal tissues subjects them to central and peripheral immune tolerance, resulting in weaker T cell responses and a higher risk of off-target toxicity when targeted by immunotherapy [25,30]. In contrast, tumor-specific neoantigens (TSA), also referred to as mutated tumor-specific antigens, arise from somatic mutations unique to malignant cells, including point mutations, insertions, deletions, or gene fusions [26,27]. These antigens are absent from the normal proteome and therefore bypass central tolerance mechanisms, eliciting robust cytotoxic T lymphocyte responses and minimal autoimmune effects [25]. Neoantigens represent the cornerstone of modern personalized immunotherapies, as demonstrated by the encouraging clinical activity of mRNA-based vaccines in early-phase trials and the progress of adoptive T cell therapies targeting these epitopes in melanoma and other cancers [26,27]. Recent proteogenomic investigations have challenged the prevailing assumption that immune recognition in melanoma primarily derives from mutated neoantigens [31]. A landmark study by Apavaloaei et al. analyzed over 500 melanoma samples and revealed that only ~1% of presented tumor antigens were mutation-derived, whereas the vast majority originated from unmutated genomic regions—either aberrantly expressed tumor-specific antigens or lineage-specific antigens—often derived from genomic regions that are normally noncoding or transcriptionally silent but become aberrantly transcribed and translated in cancer [31]. These findings suggest that unmutated tumor antigens, long overlooked due to presumed immune tolerance, may contribute substantially to spontaneous and therapy-induced antitumor immunity and represent promising targets for next-generation vaccine strategies [25,27]. The emerging consensus is that an optimal vaccine design for melanoma may integrate both mutated neoantigens, which ensure high specificity, and selected unmutated tumor antigens, which are frequently shared among patients and potentially broaden population coverage [26]. Leveraging high-throughput sequencing, proteogenomics, and bioinformatic algorithms to identify and prioritize these epitopes is essential for developing personalized yet scalable mRNA vaccines capable of addressing tumor heterogeneity while minimizing off-target immune effects [27].

### 2.2. Personalized vs. Off-the-Shelf

The design of mRNA cancer vaccines for melanoma can follow two fundamentally distinct strategies: fully personalized neoantigen-based formulations or “off-the-shelf” vaccines built on shared antigens [3,26]. Personalized vaccines rely on rapid sequencing of a patient’s tumor to identify somatic mutations, predict immunogenic epitopes through bioinformatic algorithms, and incorporate these epitopes into individualized constructs [32]. This approach maximizes specificity and minimizes off-target toxicity because the encoded antigens are absent from healthy tissues, thereby bypassing central immune tolerance and inducing potent cytotoxic T cell responses [26]. Clinical studies, most notably the randomized phase IIb KEYNOTE-942 trial in patients with completely resected stage III/IV high-risk melanoma, demonstrated that the addition of the personalized neoantigen vaccine mRNA-4157 (V940) to pembrolizumab significantly prolonged recurrence-free survival (RFS) compared with pembrolizumab alone (HR 0.561, 95% CI 0.309–1.017; 18-month RFS 79% vs. 62%). The 3-year update confirmed sustained benefit (2.5-year RFS 74.8% vs. 55.6%) and improvement in distant metastasis-free survival (DMFS), supporting the translational potential of individualized vaccines for precision oncology [3]. Conversely, off-the-shelf vaccines employ antigens that are shared across patients, such as tumor-associated antigens or lineage-restricted proteins like MART-1 and tyrosinase [33]. These vaccines offer logistical advantages: they can be manufactured at scale, distributed broadly, and administered without the delays inherent to bespoke sequencing and manufacturing pipelines [33]. However, because many shared antigens are also expressed at low levels in normal tissues, immune tolerance may limit the magnitude of the T cell response, and there is an inherent risk of autoimmune toxicity when targeting antigens with partial normal tissue expression [26]. Innovative strategies, such as the allogeneic cell-based vaccine VACCIMEL, attempt to bridge this gap by providing a broad repertoire of shared melanoma antigens while also promoting epitope spreading to patient-specific neoantigens. This dual mechanism makes VACCIMEL innovative, as it effectively combines features of both off-the-shelf and personalized vaccine approaches [34]. Emerging hybrid models further blur the boundary between these paradigms. Universal mRNA vaccine platforms encoding panels of highly recurrent neoantigens, identified through large-scale genomic profiling of melanoma, aim to capture the breadth of patient-specific immunity while retaining manufacturing efficiency characteristic of off-the-shelf products [33]. Advances in predictive modeling and artificial intelligence are critical to these efforts, enabling accurate prioritization of epitopes that maximize population coverage without compromising immunogenicity [32]. As both strategies mature, the field is converging toward flexible vaccine architectures capable of integrating personalized elements into scalable manufacturing frameworks, potentially transforming melanoma immunotherapy from highly bespoke interventions to broadly deployable precision treatments [3].

### 2.3. Tumor Antigens, Neoantigens, Costimulatory Molecules

**Figure 2:**
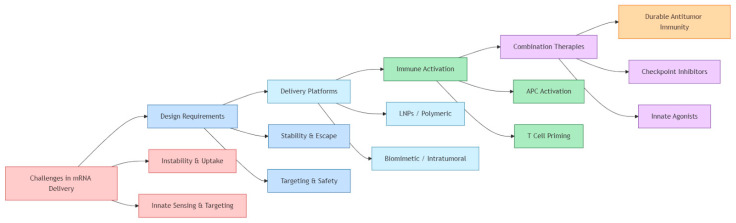
Conceptual flowchart outlining the major challenges and requirements in the delivery of mRNA vaccines, and their downstream immunological consequences. Starting from intrinsic delivery barriers—including mRNA instability, limited cellular uptake, and unintended activation of innate immune sensors—the design of efficient mRNA therapeutics must address key requirements for stability, targeted delivery, and endosomal escape. These challenges are met through diverse delivery platforms such as LNPs, polymeric carriers, and emerging biomimetic or intratumoral approaches. Effective delivery leads to antigen-presenting cell (APC) activation and efficient T cell priming, both of which are essential for eliciting robust adaptive immune responses. When integrated with combination therapies such as checkpoint inhibitors or innate immune agonists, these strategies culminate in durable antitumor immunity [38].

The performance of mRNA vaccines is dictated by the fine-tuning of multiple structural and sequence elements that collectively govern stability, translational efficiency, and immunogenicity [35]. Codon optimization within the open reading frame is a cornerstone of this process; aligning codon usage with the host transfer RNA pool minimizes ribosomal stalling and enhances protein yield [36]. Beyond codon frequency, recent computational frameworks, such as LinearDesign, integrate codon selection with RNA secondary structure optimization, producing constructs with markedly improved half-life and antigen expression compared with conventional synonymous optimization strategies [36]. Chemical modification of nucleosides represents another critical layer of refinement. Incorporation of analogues such as N^1^-methylpseudouridine or 5-methylcytidine reduces recognition by innate immune sensors like RIG-I and MDA5, mitigating undesired interferon responses while simultaneously increasing translational output [35]. Optimal modification levels are required, as excessive dampening of innate sensing may reduce the adjuvant-like properties that contribute to vaccine efficacy [35]. The untranslated regions flanking the coding sequence exert profound influence on translation kinetics and transcript stability. The five-prime untranslated region facilitates ribosome recruitment, where human-derived sequences such as β-globin 5′ UTR have been empirically shown to support superior translational efficiency compared to other candidates [37]. The three-prime untranslated region, often incorporating stabilizing motifs like α-globin or mitochondrial signals, contributes to transcript longevity and synergizes with polyadenylation in promoting mRNA circularization and efficient translation [37]. Capping of the transcript with a seven-methylguanosine structure in the Cap 1 configuration is indispensable for ribosome binding and protection against exonuclease activity, while the poly(A) tail length—commonly optimized around 100–120 nucleotides—serves to stabilize the mRNA and regulate translation initiation (Figure 2) [35].

Emerging evidence suggests that both tail length and base composition can modulate decay rates, underscoring the need for precise control during in vitro transcription and purification [37]. Collectively, these design parameters must be integrated rather than optimized in isolation. Advances in bioinformatics and machine learning are now enabling multi-objective optimization pipelines that consider codon usage, structural motifs, and chemical modifications concurrently, facilitating the generation of high-performance mRNA constructs tailored for oncology vaccine applications [36].

## 3. Delivery Challenges and Requirements

### 3.1. Stability, Uptake, Endosomal Escape, Targeting Lymph Nodes or Tumor

**Figure 3:**
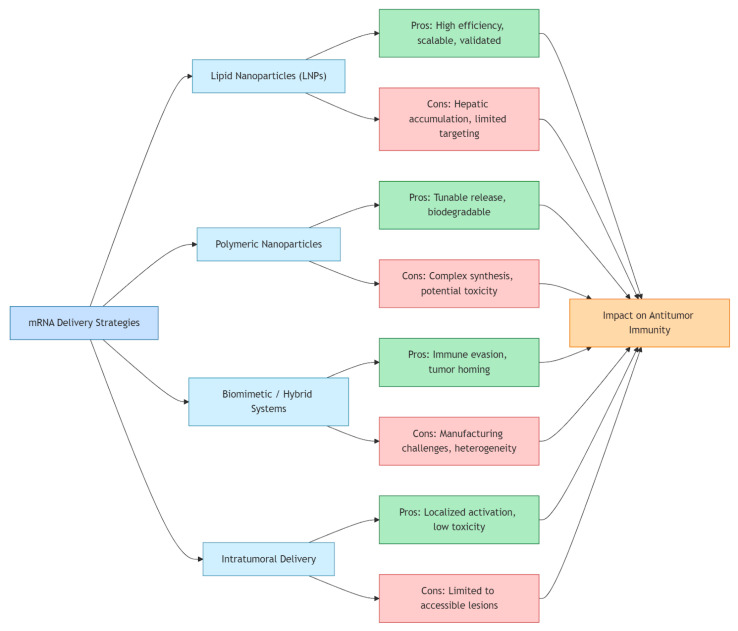
Overview of delivery strategies for mRNA cancer vaccines in melanoma. The figure illustrates four major platforms used to enhance mRNA delivery to tumor or immune cells: LNPs, polymeric nanoparticles, biomimetic or hybrid carriers, and intratumoral delivery methods. LNPs remain the clinical standard, whereas polymeric and biomimetic systems offer tailored release kinetics and immune targeting capabilities. Intratumoral delivery enables localized antigen expression, boosting immune infiltration and reducing systemic toxicity. Each approach addresses specific challenges related to RNA stability, cellular uptake, and immune activation, contributing to the evolving landscape of melanoma immunotherapy.

The therapeutic success of mRNA vaccines in melanoma is intrinsically linked to the efficiency and precision of their delivery systems [39,40]. The naked mRNA molecule is highly labile and prone to rapid degradation by extracellular ribonucleases, and its anionic nature prevents spontaneous cell membrane penetration [41]. Furthermore, unshielded mRNA can trigger potent innate immune activation through endosomal and cytosolic pattern recognition receptors such as Toll-like receptors 3, 7, and 8 or RIG-I-like receptors, potentially reducing protein translation and contributing to systemic inflammatory toxicity [39]. Overcoming these barriers requires advanced formulations capable of protecting the transcript, promoting cellular uptake, and ensuring endosomal escape into the cytoplasm where antigen expression occurs [40,41]. LNPs have emerged as the leading non-viral carriers for mRNA vaccines, owing to their modularity, scalability, and proven clinical safety [41]. Composed of ionizable lipids, cholesterol, helper phospholipids, and polyethylene glycol-lipids, LNPs encapsulate the mRNA and undergo endocytosis upon cellular contact [39]. Acidification of the endosome protonates ionizable lipids, facilitating endosomal escape through membrane destabilization [41]. Design refinements—including altering lipid headgroup chemistry, incorporating biodegradable backbones, and modulating particle size—have improved biodistribution and reduced hepatotoxicity, which is critical for extra-hepatic targeting such as melanoma lesions [39]. Alternative nanoparticle systems are actively explored to address the limitations of LNPs, particularly their preferential hepatic accumulation. Polymeric nanoparticles and dendrimer-based carriers have shown in preclinical studies the capacity to enhance tumor selectivity and modulate immune responses. In parallel, biomimetic approaches such as exosome- or membrane-derived platforms are being explored as emerging strategies, although evidence in melanoma remains limited [39,40,42,43]. These systems can be engineered to co-deliver mRNA with adjuvants such as Toll-like receptor agonists or cytokines, amplifying dendritic cell activation and adaptive immune priming [44]. Intratumoral administration represents another innovative strategy to bypass systemic barriers and exploit the immunological microenvironment of melanoma [45]. Direct injection of LNP-formulated mRNA into tumors enables localized antigen expression within malignant and stromal cells, leading to robust local immune activation and epitope spreading [44]. Preclinical models demonstrate that intratumoral delivery of mRNA encoding tumor antigens or cytokines not only suppresses local tumor growth but also induces systemic immune memory capable of controlling distant metastases [45]. Moreover, combining intratumoral mRNA vaccination with immune checkpoint inhibitors has shown synergistic efficacy, overcoming T cell exhaustion and enhancing response rates in otherwise refractory melanoma [44]. Collectively, these delivery innovations—ranging from chemical optimization of LNPs to next-generation biomimetic carriers and intratumoral administration—form the backbone of translational strategies to maximize the therapeutic index of mRNA vaccines in melanoma (Figure 3) [39,40]. As manufacturing and targeting technologies evolve, these platforms are likely to expand beyond melanoma to other solid tumors characterized by immunogenic mutational landscapes [41].

### 3.2. Lipid Nanoparticles (LNPs)

Lipid nanoparticles have emerged as the most advanced non-viral carriers for mRNA vaccines due to their modular composition, scalability, and established clinical safety [46,47]. A typical LNP comprises four main components: an ionizable cationic lipid, which condenses the negatively charged mRNA and facilitates endosomal escape; cholesterol, which stabilizes the lipid bilayer; helper phospholipids, which support membrane fusion; and polyethylene glycol-lipids, which improve colloidal stability and circulation half-life [46,48]. The physicochemical properties of each component—including pKa of the ionizable lipid, chain length, and saturation—profoundly influence biodistribution, cellular uptake, and immune activation [49,50]. Recent studies have focused on refining these parameters to enhance therapeutic index in oncology [47,51]. A 2025 Nature Communications study demonstrated that integrating LNPs into a microgel matrix (LiNx system) significantly improved lymph node recruitment, elicited balanced Th1/Th2/Th17 responses, and achieved potent antitumor immunity with a single intramuscular dose in murine melanoma models [52]. Another original work in Angewandte Chemie engineered mannose-functionalized LNPs (STLNPs-Man) that selectively targeted dendritic cells, boosting antigen presentation and reducing off-target toxicity, thereby providing a rational platform for melanoma vaccines [53]. Beyond structural optimization, functional programmability has emerged as a major innovation. New LNP frameworks incorporate responsive elements that adapt to the tumor microenvironment, such as pH-sensitive lipids or biodegradable backbones, enabling precise release profiles and minimizing hepatic accumulation—a key limitation of first-generation LNPs widely used for COVID-19 vaccines [49,50]. Furthermore, research on biodegradable LNPs for Cas9 mRNA delivery in melanoma models underscores their versatility for co-delivery of gene-editing components alongside antigen-encoding mRNA, opening avenues for in situ modulation of tumor immunogenicity [54]. These advances collectively highlight a paradigm shift: from generic mRNA delivery vehicles toward precision-engineered nanocarriers capable of targeted biodistribution, controlled release, and synergistic integration with immunomodulators [48,55]. Such next-generation LNPs not only enhance antigen expression but also shape the quality and durability of the antitumor immune response, positioning them as central to the clinical maturation of mRNA vaccines in melanoma [47,51].

### 3.3. Polymeric Nanoparticles

Polymeric nanoparticles represent a versatile and highly tunable platform for mRNA delivery, offering distinct advantages over lipid-based systems in terms of structural diversity, biodegradability, and potential for controlled release [56,57]. These carriers can be engineered from synthetic or natural polymers—including poly(lactic-co-glycolic acid) (PLGA), poly(beta-amino esters), and poly(ethylene imine)—which can be functionalized to enhance mRNA condensation, protection from nucleases, and targeted cellular uptake [58,59]. Importantly, the chemical modularity of polymers allows fine control of particle size, surface charge, and degradability, enabling precise adaptation to tumor microenvironments [57,59]. Recent advances have demonstrated significant preclinical efficacy of polymer-based mRNA vaccines in oncology [60]. A 2023 study in PNAS reported biodegradable lipophilic polymeric nanoparticles capable of delivering mRNA encoding tumor antigens to murine melanoma models, resulting in potent cytotoxic T lymphocyte activation and marked tumor regression without systemic toxicity [60]. These findings underscore the potential of biodegradable polymers to serve as clinically relevant alternatives to lipid nanoparticles for cancer vaccines [60]. Similarly, Nature Communications in 2024 described a polymeric nanoliposomal system targeting lymphoid and myeloid cells, which enhanced lymph node accumulation and improved antigen-specific immune priming in vivo, a feature particularly advantageous for melanoma vaccines where robust T cell activation is essential [58]. Beyond passive delivery, novel polymeric formulations are being designed to incorporate immunostimulatory cues [59]. For instance, a study introduced polymers conjugated with STING agonists, enabling simultaneous delivery of mRNA and innate immune activation [57]. This dual-function approach promoted dendritic cell maturation and sustained T helper 1-biased responses, offering a promising strategy to overcome the immunosuppressive tumor microenvironment that limits vaccine efficacy [58]. Collectively, polymeric nanoparticles expand the design space for mRNA vaccine delivery beyond lipid-based systems, combining high customizability with emerging bioactivity [56]. Their capacity for targeted, controlled, and immune-enhancing delivery positions them as strong candidates for next-generation melanoma vaccines, especially when integrated with combination immunotherapies [57].

### 3.4. Biomimetic and Hybrid Systems

Biomimetic and hybrid nanoparticle platforms are emerging as next-generation strategies for mRNA vaccine delivery, aiming to combine the structural advantages of synthetic carriers with the immunological benefits of biological components [61,62]. Biomimetic nanoparticles frequently employ natural membranes—derived from erythrocytes, platelets, or tumor cells—to cloak synthetic cores, conferring immune evasion, prolonged circulation, and selective homing to tumors or lymphoid tissues [63,64]. This strategy capitalizes on the inherent surface proteins and ligands of the source cells, which can facilitate enhanced antigen presentation and promote interactions with antigen-presenting cells such as dendritic cells [62,63]. Hybrid systems extend this concept by integrating multiple functionalities into a single platform [61]. For instance, recent work has developed hybrid nanovaccines that fuse tumor cell membrane vesicles with polymeric or lipidic cores encapsulating mRNA, yielding particles that simultaneously present tumor-associated epitopes on their surface while encoding complementary neoantigens internally [61,65]. In preclinical melanoma models, such constructs induced both humoral and cytotoxic T cell responses, achieving superior tumor control compared with conventional lipid nanoparticles [65]. A major advantage of biomimetic systems lies in their potential to modulate the tumor microenvironment [64]. By displaying immune co-stimulatory molecules or chemokines on the nanoparticle surface, these carriers can actively recruit and activate effector lymphocytes at the tumor site, overcoming local immune suppression [62]. Moreover, hybrid formulations can co-deliver adjuvants—such as Toll-like receptor agonists or stimulator of interferon genes (STING) activators—together with mRNA, orchestrating synergistic innate and adaptive immune responses [61]. While these platforms remain largely preclinical, they represent a promising frontier in mRNA vaccine design [63]. Their unique ability to emulate the complexity of biological interactions, while retaining the scalability of synthetic systems, may enable personalized yet broadly applicable cancer vaccines [62]. Continued optimization of membrane source, hybridization techniques, and manufacturing protocols will be critical to translating these technologies to clinical melanoma immunotherapy [64].

### 3.5. Intratumoral Delivery Strategies

Intratumoral administration of mRNA vaccines represents a promising strategy to overcome systemic delivery barriers and exploit the immunologically active tumor microenvironment [66,67]. Direct injection into the tumor bypasses hepatic sequestration and systemic clearance, allowing localized antigen expression within malignant and stromal cells [68,69]. This localized delivery can induce potent in situ vaccination effects, characterized by enhanced dendritic cell activation, increased infiltration of cytotoxic CD8^+^ T lymphocytes, and epitope spreading that extends immune recognition beyond the encoded antigen [67,70]. Recent preclinical studies have demonstrated the therapeutic potential of intratumoral mRNA delivery in melanoma models [45,71]. For example, intratumoral injection of mRNA encoding tumor antigens or immunostimulatory cytokines resulted in substantial tumor growth delay and, when combined with immune checkpoint blockade, achieved durable systemic responses capable of controlling distant lesions—an observation consistent with an abscopal effect, where localized immune activation propagates to systemic tumor control [67,70,72]. These findings highlight the synergy between localized antigen expression and systemic immune modulation, offering a compelling rationale for integrating intratumoral vaccination into multimodal immunotherapy regimens [66,73]. In addition to antigen-encoding constructs, intratumoral strategies enable co-delivery of adjuvants or agents targeting immune suppressive components of the tumor microenvironment, such as myeloid-derived suppressor cells or regulatory T cells [68,69]. Hybrid approaches, including oncolytic viruses engineered to deliver mRNA payloads or nanoparticles designed for pH-triggered release, further expand the functional versatility of intratumoral vaccines [67,71]. However, practical challenges remain, including heterogeneous tumor accessibility, variable injection feasibility across metastatic sites, and the need for imaging-guided administration protocols to ensure reproducible dosing [69,73]. Despite these limitations, intratumoral delivery has distinct translational advantages. By concentrating antigen expression at the tumor site, it reduces systemic toxicity and permits lower total mRNA doses, potentially lowering manufacturing costs [74,75]. As clinical studies advance, intratumoral vaccination is poised to complement systemic mRNA immunotherapies, particularly in tumors like melanoma that are accessible to direct injection and characterized by a highly immunogenic mutational landscape [71,73].

## 4. Clinical and Preclinical Evidence

### 4.1. Key Trials (e.g., Moderna mRNA-4157/V940, BioNTech FixVac)

Preclinical explorations of mRNA vaccines in melanoma consistently reveal a capacity to trigger strong, mutation-specific T cell responses that extend well beyond the initial antigenic targets [76,77]. In murine models, personalized constructs encoding neoantigens derived from tumor exome sequencing provoked durable cytotoxic activity and immune memory, findings that laid the groundwork for subsequent human trials and justified rapid clinical translation [76,77]. Momentum in the clinic has been led by the phase 2b KEYNOTE-942 study, where the personalized vaccine mRNA-4157 (V940, Moderna) was paired with pembrolizumab as adjuvant therapy in patients with resected high-risk melanoma [77,78]. Updated analyses demonstrated a pronounced reduction in the risk of recurrence or death—44% at two years and nearly 50% at three—while also improving distant metastasis-free survival [77,78]. Importantly, this benefit came without unexpected safety concerns, strengthening the rationale for integrating bespoke mRNA vaccines into standard checkpoint blockade regimens [78]. In parallel, BioNTech has advanced the FixVac approach with BNT111, a vaccine targeting a defined set of shared melanoma antigens (NY-ESO-1, MAGE-A3, tyrosinase, TPTE) [79,80]. Combined with cemiplimab, this off-the-shelf construct produced meaningful gains in overall response rates in stage III/IV unresectable melanoma and earned Fast Track status from the U.S. Food and Drug Administration [81,82]. The trial’s results underline that fixed-antigen vaccines, while less individualized, can still achieve clinically significant activity and are inherently easier to scale for broader patient populations. Together these findings reflect two converging strategies: personalized vaccines that exploit the unique mutational landscape of each tumor, and shared-antigen vaccines that prioritize manufacturing feasibility and rapid deployment [77,82]. Both are reshaping therapeutic options in melanoma and provide complementary templates for integration with emerging combination immunotherapies [78,83].

### 4.2. Intratumoral vs. Systemic Delivery Outcomes

Direct intratumoral administration of mRNA vaccines achieves a pharmacodynamic profile that systemic routes struggle to replicate [84]. In murine melanoma, local injection of lipid nanoparticle–encapsulated mRNA results in robust antigen expression within both malignant and stromal cells, accompanied by a surge of CD8^+^ T lymphocytes infiltrating the tumor bed and draining lymph nodes [45]. This localized immune activation translates into slower tumor progression and extended survival, even with single-dose regimens, while systemic dosing of the same construct elicits weaker tumor infiltration despite similar serum cytokine levels [44,45]. Beyond antigen expression, intratumoral delivery reshapes the tumor microenvironment. mRNA constructs encoding immunostimulatory cytokines such as interleukin-12 have been shown to convert suppressive myeloid niches into pro-inflammatory hubs, diminishing regulatory T cells and driving interferon-gamma production [44,85]. These effects are spatially restricted to the tumor site, minimizing systemic toxicity and circumventing dose-limiting inflammatory events observed with intravenous administration [85]. Systemic delivery, by contrast, retains relevance for disseminated disease, providing the possibility of priming immune responses across multiple metastatic sites simultaneously [84]. However, systemic mRNA vaccines are prone to hepatic sequestration and rapid clearance, necessitating higher doses and advanced nanoparticle engineering to achieve tumor targeting [44]. The trade-off between localized potency and systemic reach is shaping ongoing clinical trial designs, where hybrid strategies—such as priming with systemic dosing followed by intratumoral boosting—are beginning to emerge [84]. These comparative insights underscore that route of administration is not merely logistical but immunologically decisive [84]. Determining when to exploit the focused, high-intensity response of intratumoral delivery versus the broader, whole-body coverage of systemic approaches remains central to optimizing mRNA vaccine regimens for melanoma [84].

## 5. Integration with Combination Therapies

Incorporating cytokines or innate immune agonists into mRNA vaccine formulations has emerged as a way to overcome immune resistance and amplify antitumor responses [86]. Local co-delivery of mRNA encoding interleukin-12, interleukin-7, and interferon-α in melanoma models produced a striking shift toward Th1 polarization, with dense CD8^+^ infiltration and long-term tumor control that surpassed checkpoint blockade alone [87,88]. These effects were not confined to the injected lesion: epitope spreading supported systemic immunity capable of controlling distant metastases [87,89]. Nanoparticle strategies that package antigen mRNA together with cytokines achieved similar gains while reducing systemic toxicity [90]. An LNP platform encoding both tumor antigen and IL-12, described in Science Immunology elicited robust cytotoxic T cell responses without triggering the dose-limiting inflammation often seen with recombinant cytokine therapies [90]. Such dual-payload constructs blur the line between vaccine and adjuvant, concentrating immune activation where it is most needed [91]. Synthetic adjuvants provide an additional layer of control. Lipid-linked Toll-like receptor 7/8 agonists or amphipathic peptides incorporated into the mRNA carrier enhance dendritic cell activation and cytokine secretion, creating a pro-inflammatory milieu that accelerates T cell priming [91]. Beyond nucleic acid-based approaches, preclinical studies in various cancer models have shown that selected small-molecule immunomodulators can increase tumor immunogenicity [92,93], suggesting broader opportunities to integrate diverse immune-potentiating agents into future mRNA vaccine designs. Reviews of these approaches highlight their potential to reshape vaccine immunobiology—moving beyond the idea of mRNA as a simple antigen blueprint toward multifunctional constructs that direct and sustain the desired immune phenotype [86]. Together, these findings suggest that co-delivering cytokines or innate agonists with mRNA vaccines is more than an incremental improvement; it is a strategic redesign of how cancer vaccines engage the immune system, offering a route to deeper and more durable remissions in melanoma [86,94].

## 6. Future Directions

Progress in mRNA vaccine technology for melanoma is accelerating toward increasingly individualized strategies [95,96]. Advances in genomic profiling and rapid sequencing now allow comprehensive mapping of tumor neoantigens in days rather than weeks, opening the door to fully bespoke vaccines tailored to each patient’s mutational landscape [96,97]. Machine learning tools trained on large immunopeptidomic datasets are beginning to predict which epitopes will elicit durable T cell responses, outperforming traditional binding-affinity algorithms and supporting the rational design of next-generation constructs [95,98]. Artificial intelligence is also reshaping other aspects of vaccine engineering, from codon optimization to the selection of untranslated regions that fine-tune translation efficiency and stability [95,96]. These computational frameworks are increasingly integrated into automated manufacturing pipelines, bridging the gap between in silico design and clinical-grade production [96,97]. Scalability remains a pivotal challenge. While personalized vaccines promise unmatched specificity, they demand rapid, decentralized production and robust quality control frameworks [99]. Emerging modular manufacturing platforms—capable of synthesizing multiple mRNA constructs in parallel—aim to reduce cost and turnaround time [99]. Regulatory agencies are beginning to adapt as well, drafting guidance for individualized mRNA therapeutics that diverge from conventional batch-release paradigms [99]. Looking ahead, hybrid strategies may dominate: partially personalized vaccines built on libraries of recurrent melanoma neoantigens, refined by AI to match individual tumor profiles, and manufactured at scale [96,97]. This convergence of personalization and standardization—coupled with improved delivery platforms and combination regimens—signals a future where mRNA vaccines move beyond proof-of-concept into routine oncology practice [95,96].

## 7. Conclusions

mRNA vaccines have moved from experimental concept to clinical trials in melanoma, propelled by rapid innovations in antigen selection, delivery platforms, and combination strategies. Personalized constructs like mRNA-4157 demonstrate that tailoring vaccines to a patient’s unique mutational profile can substantially reduce recurrence risk when layered onto checkpoint blockade, while fixed-antigen approaches such as BNT111 provide scalable alternatives for broader populations. Parallel advances in delivery—from refined lipid nanoparticles to biomimetic hybrids and intratumoral administration—are dismantling long-standing barriers of stability, biodistribution, and immune activation. The field now faces a dual challenge: sustaining the pace of innovation while addressing practical realities of manufacturing, regulation, and equitable access. Artificial intelligence and high-throughput sequencing promise to streamline epitope discovery and vaccine design, but translating these capabilities into routinely deployable therapies will require harmonized regulatory frameworks and global infrastructure for rapid production. The convergence of these technologies suggests a future in which mRNA vaccines are not adjuncts but central pillars of melanoma immunotherapy, integrated seamlessly with checkpoint inhibitors, cytokine modulators, and emerging cellular therapies.

## Data Availability

All data relevant to the study are included in the article.
